# Self-Reported Psychosomatic Complaints In Swedish Children, Adolescents, and Young Adults Living in Rural and Urban Areas: An Internet-Based Survey

**DOI:** 10.2196/publichealth.5902

**Published:** 2017-03-07

**Authors:** Katarina Laundy Frisenstam, Matilda van den Bosch, Yun Chen, Peter Friberg, Walter Osika

**Affiliations:** ^1^ The Sahlgrenska Academy and University Hospital Department of Molecular and Clinical Medicine/Clinical Physiology University of Gothenburg Gothenburg Sweden; ^2^ School of Population and Public health The University of British Columbia Vancouver, BC Canada; ^3^ Department of Forest and Conservation Sciences The University of British Columbia Vancouver, BC Canada; ^4^ Center för Social Sustainability, Department for Neurobiology, Care Sciences and Society Department of Clinical Neuroscience Karolinska Institutet Stockholm Sweden

**Keywords:** adolescents, children, environmental risk, psychosomatic, urban living, Internet

## Abstract

**Background:**

Frequencies in reported psychosomatic illnesses have increased in Sweden among children, adolescents, and young adults. Little is known about demographic differences in self-reported psychosomatic complaints, such as between urban and rural areas, and whether surveys launched on the Internet could be a useful method in sampling such data.

**Objectives:**

This study examines the connection between psychosomatic illnesses and demographics in Swedish children and youth. The feasibility of using the Internet to gather large amounts of data regarding psychosomatic complaints in this group is another major objective of this study.

**Methods:**

A cross-sectional study using 7 validated questions about psychosomatic health, were launched in a controlled way onto a recognized Swedish Internet community site, which targeted users 10 to 24 years of age. The subjects were able to answer the items while they were logged in to their personal domain. The results were analyzed cross-geographically within Sweden.

**Results:**

In total, we received 100,000 to 130,000 individual answers per question. Subjects of both sexes generally reported significantly higher levels of self-reported psychosomatic complaints in major city areas as compared with minor city/rural areas, even though the differences between the areas were small. For example, 12.00% (4472/37,265) of females in minor regions reported always feeling tense, compared with 13.80% (3156/22,873) of females in major regions (*P*<.001). In males, the answer pattern was similar, 16.40% (4887/29,801) in major regions versus 15.60% (2712/17,386) in minor regions, (*P*=.006). Females reported significantly higher frequencies of psychosomatic complaints than males (*P*<.001).

**Conclusions:**

In subjects aged 10 to 24 years, higher levels of psychosomatic complaints appear to correlate with living in major city areas in comparison with minor city/rural areas. Surveys launched on the Internet could be a useful method in sampling data regarding psychosomatic health for this age group.

## Introduction

Children, adolescents, and young adults in Sweden frequently report mental health issues [1,2]. The mental health issues we are referring to in this Swedish population are most commonly complaints about perceived stress and psychosomatic symptoms. It is speculated that current lifestyles, environments, and societal demands, with increased exposure to stress, play a role in the changing patterns of mental health problems in this population. With less opportunity for physical activity, play, and recovery, stress reactions may become chronic, which increases the risk for psychosomatic complaints and mental disorders [3-6].

The development from chronic stress to psychosomatic symptoms and eventually disease depends on multiple factors, such as genetic vulnerability [7], socioeconomic situation [8], adverse or stressful life events and the timing of such [9], disturbances in important relationships, and school performance, which all fit into a bio-psycho-social model [10], as well as the concept of the developmental origin of health and disease [11].

In addition, the surrounding environment has an impact on disease development [12]. For example, while cities are centers of wealth creation, culture, and innovation, they are also associated with adverse health outcomes that are less prevalent in rural areas. The “unhealthy” urban environment may be due to increased exposure to harmful pollutants and denser, more stressful living. Previous research has shown that living in an urban environment is associated with an increased prevalence of mental health disorders, such as schizophrenia and depression [13-16]. This has been partly ascribed to higher levels of social stress in cities as compared with in rural areas. Lederbogen et al [17] recently showed that both city upbringing and current city living were associated with increased amygdala activity, which, among other functions, signals negative affect, stress, and environmental threat. Increased amygdala activity has been strongly implicated in anxiety disorders and depression [13]. Assuming that a rural population is more exposed to nature, this would confirm the increasing evidence that natural (green and blue) environments have a restorative influence, resulting in decreased stress levels and positive effects on mental health [18-20]. These benefits may also be gained by moving to urban green areas [21,22]. A recent study, investigating symptoms of depression, demonstrated that walking in nature decreased both self-reported rumination and neural activity in the subgenual prefrontal cortex (associated with stress and negative thought activity), while such changes were not found after a walk in an urban environment [23]. In particular, children and young people seem to benefit the most from natural environments with improvements in cognitive and behavioral function and development [24,25]. For example, this may be due to trees’ contribution to reduction of toxic environmental exposures, such as traffic-related air pollution and noise [26-29], which are risk factors for poor development and mental health problems [30-32]. These environmental risks are less pronounced in rural environments. In an increasingly urbanized world, these issues are important to explore. Thus, genetic, social, and physical environmental factors interact in the development of stress and psychosomatic reactions in such a way that in due course they may influence children's well-being and ill health, with salient implications also for development of future health and disease in the life course perspective [33].

In a previous Internet-based study, we have shown that stress and psychosomatic health complaints are common in children, adolescents, and young adults. Older teenage females (16-18 years of age) had the highest levels of complaints, and both sexes reported a slightly worse self-perceived general health status in 2010 than in 2007 [2]. However, this study did not investigate the impact of living environment on self-reported psychosomatic complaints. Previous studies have found a positive environmental impact on mental health, with a protective effect of natural, less urbanized areas, but most of these studies are performed on adult populations [33].

The purpose of the present study was to examine self-reported psychosomatic complaints among children and adolescents, to unravel potential differences with regard to these complaints depending on sociodemographic factors and residential environment in Sweden. Our hypothesis is that psychosomatic complaints are more prevalent among the young in urban areas and more frequent in females

## Methods

### Participants

The use of Internet has the benefit of providing a high number of respondents in all categories concerning age, sex, and geographical region. We recruited 130,000 study participants by convenience sampling through the website LunarStorm. LunarStorm's website was one of the first social Web communities to be established in Sweden. At the time of our sampling (2005), LunarStorm was the largest Internet community in Sweden. It had 1.3 million active members and approximately 360,000 unique visitors per day who spent approximately 40 minutes per visit on the site (TNS Gallup/Red Measure, Nielsen/Net Ratings). Among 15- to 20-year olds in Sweden by that time, 83% were LunarStorm members, and 25 of 30 pupils in every secondary school class were connected (Lunarworks AB/SCB). Of members, 53% were females.

### Measures and Design

To assess psychosomatic complaints among the participants we used the well-established Psychosomatic Problems Scale (PBS) [1,34,35]. The scale has been tested for reliability by Hagquist, using Rasch analysis, and has sound psychometric properties as a whole [1,34,35]. The Swedish National Board of Health and Welfare use it in studies of children and adolescents and their psychosomatic health and wellbeing. PBS is a composite measure of subjective health experienced during the last 6 months, encompassing the following items (which were delivered in question form): ‘difficulty concentrating,’ ‘difficulty sleeping,’ ‘suffers from headaches,’ ‘suffers from stomach aches,’ ‘feels tense,’ ‘poor appetite,’ and ‘feels low’. The 6 response categories to the items were: ‘don't know’; ‘no, never’; ‘no, seldom’; ‘yes, sometimes’; ‘yes, often’; and ‘yes, always.’

Our data were collected by launching the questions on 7 consecutive days in the spring of 2005. We posted 1 question per day rather than presenting the complete questionnaire on a single occasion because the Web community administrator had the experience that using long composite questionnaires substantially decreased the participation rate. However, it is therefore not possible to directly compare the results of this study with results from studies using the original composite measure. Each participant could log into their own ‘LunarStorm corner' and voluntarily choose to complete the questions in private and in their own time on the community site. These privacy and time aspects may contribute to more reliable replies [36]. All data on self-reported psychosomatic complaints from the Lunarstorm platform were sampled through a noncommercial collaboration between the research team and Lunarstorm *.*

While the use of computerized psychological assessment has increased with time, there are a limited number of validation analyses regarding Internet-based surveys among young people. However, most research to date seems to conclude that results from Internet-based recruitment corresponds well to results from more traditional administration means. In a recent study, a brief Web-based screening questionnaire for common mental disorders was validated with follow-up phone interviews, using a Diagnostic and Statistical Manual of Mental Disorders–based interview manual as a gold standard [37]. Good agreement between responses to hand out questionnaires and those administered via the Internet was demonstrated in yet another study from 2005 [38], as well as in a recent review, where most scales show high interformat reliability [39]. Internet distribution may potentially also improve participation rate, particularly so among adolescents, by avoiding certain barriers, such as putting a questionnaire in the mail [40]. Equally, it widens geographical access and facilitates the recruitment of rural populations [41].

Members saw the question after login, and only 1 answer per login was permitted and counted. Each answer was registered as unique; hence, it was not possible repeat login and reply more than once. We focused on young people aged 10 to 24 years. The age groups were differentiated as follows: 10 to 12 years (children), 13 to 16 years (adolescents), and 17 to 24 years (young adults). The percentage of LunarStorm members in these different age groups, ranged between 20% and 88%, with the highest values (>80%) being adolescents between 13 and 16 years of age.

Based on individual Internet protocol adresses, we made a geographical categorization of the subjects into 3 major city regions (Stockholm, Göteborg, and Malmö) and 18 minor cities and regions with lower population density in Sweden (Blekinge, Dalarna, Gotland, Gävleborg, Halland, Jämtland, Jönköping, Kalmar, Kronoberg, Norrbotten, Södermanland, Uppsala, Värmland, Västerbotten, Västernorrland, Västmanland, Örebro, and Östergötland). This sample was then dichotomized into ‘major city regions’ and ‘minor city/rural regions.’

The LunarStorm site did not provide data on socioeconomic background. Therefore, we added demographic data separately via Statistics Sweden, from the Swedish Living Conditions Surveys [42], and assigned to the participants on a group level determined by geographical location. This general Swedish sampling from the Swedish Living Conditions Survey was made in 2005 by interviews at home visits, and 75.10% (4277/5698) of eligible subjects were interviewed. Data on household income for 2005 was accessed separately via Statistics Sweden [43]. The data from Statistics Sweden are categorized in similar categories as ours; however, the major cities are contained within counties with additional smaller cities, towns, and villages around the large cities.

### Statistical Analysis

Each of the possible responses to each of the 7 questions in the Likert format was assigned a number: ‘no, never=1’; ‘no, seldom=2’; ‘yes, sometimes=3’; ‘yes, often=4’ and ‘yes, always=5’. Respondents answering, “don’t know” were excluded from the analysis. The response frequency was calculated and independent samples *t* tests were conducted to investigate any significant differences in mean ranks depending on geographic location (major and minor regions) or gender. Variables were tested for normal distribution by Kolmogorov-Smirnov test. Normally distributed variables are presented as means (SD). A two-sided *P*=.05 was considered as statistically significant. All statistical analyses were performed with SPSS22.0.

### Ethical Considerations

Ethical approval was obtained from the chairman of the review board. According to the ethical guidelines of the ethics board in Gothenburg, Sweden, posting questionnaires on the Internet does not require formal ethical approval from a committee. However, we choose to discuss these issues thoroughly with the chairman and received full approval.

## Results

### Participant Characteristics

**Figure 1:**
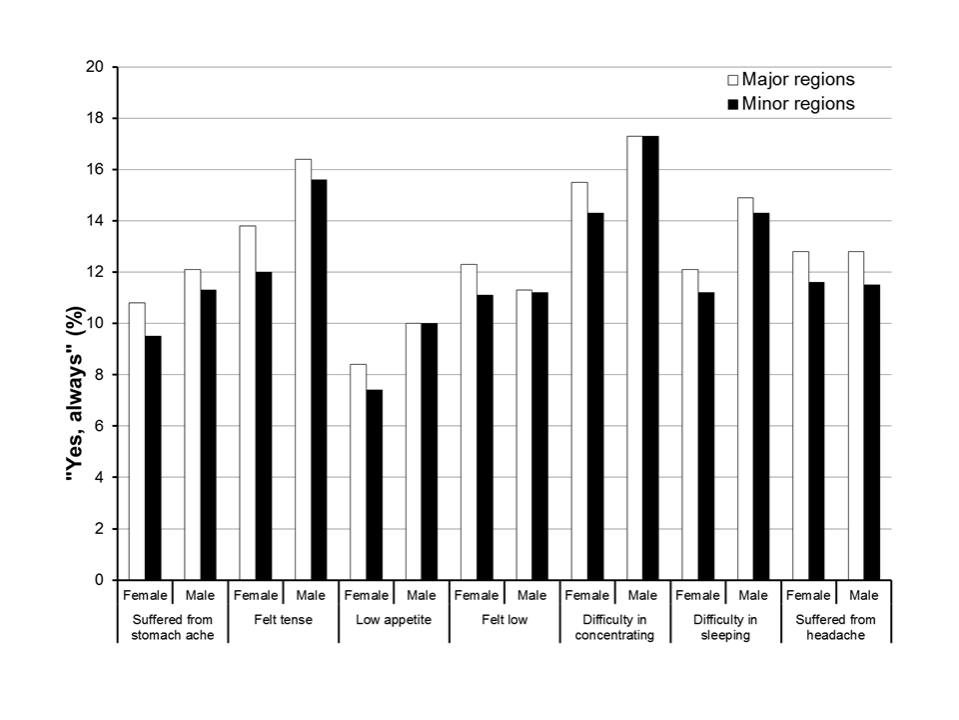
The percentage of ”Yes, always” responses to different psychosomatic complaints for 10- to 24-year-old females and males from major and minor regions of Sweden.

Females answered the questions more frequently, and also reported significantly higher frequencies of psychosomatic complaints than males on all 7 questions (*P*<.001) (Table 1). This was a consistent pattern for the majority of the questions with the exception of 3 questions in males, where there was no significant difference between the major city regions and the minor city/rural areas regarding ‘difficulty in concentrating,’ ‘low appetite,’ and ‘felt low’ (Figure 1).

### Outcomes

For all 7 questions, females from the small city/rural areas presented with better self-perceived health as compared with those from the major city areas (Table 2, Figure 1).

**Table 1:** Sex differences regarding self-reported psychosomatic complaints.

Question/alternative			Yes, always, %	Yes, often, %	Yes, some-times, %	No, seldom, %	No, never, %	Number of responders	Overall *P* value
**Suffered from stomach ache**									
	Major regions								
		Females	10.80	16.10	46.20	19.50	7.40	24,846	
		Males	12.10	6.20	31.90	27.90	22.00	16,408	<.001
	Minor regions								
		Females	9.50	16.10	46.10	20.60	7.60	41,661	
		Males	11.30	6.00	31.50	28.30	23.00	28,198	<.001
**Felt tense**									
	Major regions								
		Females	13.80	21.90	44.50	14.00	5.80	22,873	
		Males	16.40	10.90	41.70	17.80	13.20	17,386	<.001
	Minor regions								
		Females	12.00	21.10	45.40	15.50	6.10	37,265	
		Males	15.60	9.80	41.30	18.70	14.60	29,801	<.001
**Low appetite**									
	Major regions								
		Females	8.40	9.40	41.20	22.80	18.10	22,770	
		Males	10.00	5.00	28.70	23.40	33.00	15,785	<.001
	Minor regions								
		Females	7.40	8.90	41.50	23.60	18.60	36,860	
		Males	10.00	4.70	28.20	24.10	33.00	26,919	<.001
**Felt low**									
	Major regions								
		Females	12.30	27.00	47.70	9.90	3.10	31,056	
		Males	11.30	11.20	43.40	21.60	12.50	19,817	<.001
	Minor regions								
		Females	11.10	27.00	48.30	10.60	3.10	50,511	
		Males	11.20	11.50	42.90	22.30	12.20	33,409	<.001
**Difficulty in concentrating**									
	Major regions								
		Females	15.50	21.90	47.60	10.50	4.60	27,883	
		Males	17.30	14.60	44.40	14.70	9.00	20,114	<.001
	Minor regions								
		Females	14.30	21.90	48.70	10.70	4.40	46,293	
		Males	17.30	14.60	44.60	14.70	8.80	33,792	<.001
**Difficulty in sleeping**									
	Major regions								
		Females	12.10	13.10	44.10	21.80	8.80	29,488	
		Males	14.90	9.30	35.40	24.20	16.10	20,621	<.001
	Minor regions								
		Females	11.20	12.70	44.40	22.60	9.20	48,583	
		Males	14.30	8.90	35.70	24.60	16.60	34,999	<.001
**Suffered from headache**									
	Major regions								
		Females	12.80	18.60	48.90	18.50	5.30	26,494	
		Males	12.80	8.70	38.60	27.30	12.60	17,899	<.001
	Minor regions								
		Females	11.60	18.30	45.20	19.60	5.20	43,164	
		Males	11.50	8.10	38.70	28.90	12.90	30,808	<.001

**Table 2:** Percentage demographic distribution of responders across Sweden, answering 7 items of self- reported psychosomatic complaints on the web in May 2005.

Question/alternative		Yes, always %	Yes, often %	Yes, sometimes %	No, seldom %	No, never %	Number of responders	Overall *P* value	Interpretation
**Suffered from stomachache**									
	Females, major regions	10.80	16.10	46.20	19.50	7.40	24,846		
	Females, minor regions	9.50	16.10	46.10	20.60	7.60	41,661	<.001	Females in minor regions fewer complaints
	Males, major regions	12.10	6.20	31.90	27.90	22.00	16,408		
	Males, minor regions	11.30	6.00	31.50	28.30	23.00	28,198	<.001	Males in minor regions fewer complaints
**Felt tense**									
	Females, major regions	13.80	21.90	44.50	14.00	5.80	22,873		
	Females, minor regions	12.00	21.10	45.40	15.50	6.10	37,265	<.001	Females in minor regions fewer complaints
	Males, major regions	16.40	10.90	41.70	17.80	13.20	17,386		
	Males, minor regions	15.60	9.80	41.30	18.70	14.60	29,801	<.001	Males in minor regions fewer complaints
**Low appetite**									
	Females, major regions	8.40	9.40	41.20	22.80	18.10	22,770		
	Females, minor regions	7.40	8.90	41.50	23.60	18.60	36,860	<.001	Females in minor regions fewer complaints
	Males, major regions	10.00	5.00	28.70	23.40	33.00	15,785		
	Males, minor regions	10.00	4.70	28.20	24.10	33.00	26,919	.41	No difference
**Felt low**									
	Females, major regions	12.30	27.00	47.70	9.90	3.10	31,056		
	Females, minor regions	11.10	27.00	48.30	10.60	3.10	50,511	<.001	Females in minor regions fewer complaints
	Males, major regions	11.30	11.20	43.40	21.60	12.50	19,817		
	Males, minor regions	11.20	11.50	42.90	22.30	12.20	33,409	.74	No difference
**Difficulty in concentrating**									
	Females, major regions	15.50	21.90	47.60	10.50	4.60	27,883		
	Females, minor regions	14.30	21.90	48.70	10.70	4.40	46,293	.006	Females in minor regions fewer complaints
	Males, major regions	17.30	14.60	44.40	14.70	9.00	20,114		
	Males, minor regions	17.30	14.60	44.60	14.70	8.80	33,792	0.61	No difference
**Difficulty in sleeping**									
	Females, major regions	12.10	13.10	44.10	21.80	8.80	29,488		
	Females, minor regions	11.20	12.70	44.40	22.60	9.20	48,583	<.001	Females in minor regions fewer complaints
	Males, major regions	14.90	9.30	35.40	24.20	16.10	20,621		
	Males, minor regions	14.30	8.90	35.70	24.60	16.60	34,999	.006	Males in minor regions fewer complaints
**Suffered from headache**									
	Females, major regions	12.80	18.60	48.90	18.50	5.30	26,494		
	Females, minor regions	11.60	18.30	45.20	19.60	5.20	43,164	<.001	Females in minor regions fewer complaints
	Males, major regions	12.80	8.70	38.60	27.30	12.60	17,899		
	Males, minor regions	11.50	8.10	38.70	28.90	12.90	30,808	<.001	Males in minor regions fewer complaints

In the separate data sampling performed via Statistics Sweden [44], the informants answered questions regarding socioeconomic status and the results the indicated differences between urban and rural areas (the same city regions as in the Lunarstorm data sampling). The data suggest that inhabitants in the major city regions generally have better education, access to the Internet, and higher employment levels. In contrast, in the major city regions the populations are in general more economically disadvantaged.

When analyzing household income data (Table 3) it seems that although there is a generally even distribution of income across Sweden, the poorest of the adult population (rated as receiving zero income) are found in counties containing the large city areas as well as the high earners [43].

**Table 3:** Demographic distribution of population with zero income.

% 0 income	All age groups	20-24 years	25-29 years	30-34 years	35-39 years	40-44 years	45-49 years
**Urban, mean (SD)**							
	5.70 (0.87)	10.20 (2.19)	6.43 (1.43)	4.49 (1.17)	3.84 (0.87)	3.74 (0.91)	3.48 (0.88)
**Rural, mean (SD)**							
	4.13 (0.78)	7.79 (1.58)	4.63 (0.86)	2.61 (0.56)	2.07 (0.52)	2.10 (0.52)	1.91 (0.49)
*P* (*t* test)	.005	.031	.006	Not significant	<.001	<.001	<.001

## Discussion

### Principal Findings

The major finding of this study was that psychosomatic complaints were reported to a significantly higher degree in females from the major city areas as compared with the minor city/rural areas. This pattern also prevailed for males, although there was no statistically significant difference regarding the following items: difficulty in concentrating, low appetite, and felt low (Figure 1).

The higher levels of complaints in females compared with males in both area categories support and extend the results from earlier studies [1,2,8]. Similar findings, using the same questionnaire items, have been reported in other studies where youth were recruited from small regions in Sweden [1,34,35] and psychosomatic complaints were examined using the questionnaires in a manual format handed out in schools at regular intervals since 1985. To our knowledge, the current study is the first large study with more than 100,000 participants to investigate differences in psychosomatic health in relation to living in large city regions versus minor city/rural regions.

These results are in line with a study by Samanta et al [45] where rural living appeared protective against development of mental health issues among male adolescents (13-15 years), as well as with an earlier study in New Zealand, where adolescents from larger population centers reported more life event stresses than those from smaller centers [46]. The mechanisms underlying these region-related differences in psychosomatic health cannot be revealed by the current study. However, it is interesting to mention that the study by Lederbogen et al [17] shows that urban living is a risk factor for decreased social stress resilience with subsequently higher prevalence of mental disorders than rural living. There could also be regional differences in coping with mental health. Wang et al [47] recently showed that rural participants had a larger total number of visiting days, in a in a Web-based trauma intervention and visited more program modules than urban participants. With few exceptions [28], most studies on general psychiatric disorders show that the admission rates are higher in urban than in rural areas [3,4,48]. Even though the current study did not investigate specific psychiatric diagnoses, our findings are in line with the research on the effects of urbanization, especially the effect on stress-related disorders.

Several other factors are known to increase the risk for psychosomatic complaints among children and adolescents, such as housing, school system, neighborhood context, and other environmental issues [8,9,49-51]. These factors are often interrelated and the causality is complex. In general, living under poor socioeconomic conditions is strongly correlated to adverse mental and physical health outcomes [52]. In order to get complementary information on the sociodemographic background for our sample, we used data from Statistics Sweden collected in 2005 [43]. While we were unable to include these data as confounders in the analysis, due to group level reporting, the data suggest that living in 1 of the minor regions is associated with a less adverse economic situation, and with higher access to a daily newspaper than the major city regions, but it was also apparent that access to the Internet in the home, completed postsecondary education, and employment was higher in the major city regions than the minor regions. Our analysis of income shows a pattern of clusters of zero income earners being concentrated in the urban areas. In the counties containing the major cities, 0.58% of the population had exceptionally high earnings as compared with 0.20% for the rest of the country, which is pointing to some inequalities regarding income in urban areas.  

We speculate that these large socioeconomic differences in the urban areas may contribute to the ratings of higher degrees of psychosomatic complaints in our subjects living in these areas. These complementary data may have provided a partial explanation for the regional differences in self-reported psychosomatic health found in the current study. Our approach did not allow matching on an individual level for the 2 different datasets (the data collected from the website and the data from Statistics Sweden), but merely provides a spatial correlation for the major and the minor areas. However, it provides a general picture of the geographical distribution of socioeconomic issues in Sweden and how these, at least partly, overlap with the general picture of the distribution of psychosomatic complaints in respective areas.

We are unable to draw any conclusion about the causality between city living and the potentially increased risk for psychosomatic complaints from this cross-sectional study. One hypothesis is that differences in exposure to nature and green areas for recreation may account for some of the variance. Numerous studies show that access to green spaces has been associated with health benefits at both individual and neighbourhood levels [22,53,54] and that components of urban city living, such as traffic noise, air pollution, and crowds have negative effects on psychological health [55-57]. In addition, socioeconomically related health differences seem to decrease with increasing amount of green in the living area [58]. Although our data did not provide information on land use or environmental characteristics, we can assume that the major city areas are less green than the more rural areas. Sweden is a scarcely populated country and the majority of the population is concentrated to the large city areas. Thus, natural environments and less densely populated towns and cities characterize the rest of the country. The socioeconomic inequalities in urban areas are also an interesting observation that possibly contributes to the wellbeing of young people [59].

A common stressor in adults is having a low perceived degree of control over ones day to day life: it is plausible that living in an urban environment with plenty of potentially challenging outer structures, such as living in dense areas, commuting and fighting for resources on highly competitive social arenas could be stress factors also accounting for this variability in young people. Social stress and lack of control [50] could mediate the stressful effects of city life, and might account for some of the individual differences seen [17].

### Limitations

Because the data were gathered in collaboration with the LunarStorm site that did not provide information regarding specific age, socioeconomic background, ethnicity, family background, living conditions, or general wellness the analytical base of the study is limited, with restricted control for confounding variables. Some accountancy for this was taken by the additional dataset from Statistics Sweden displaying sociodemographic patterns of the populations on a regional spatial level.

The PBS was originally analyzed when all items were answered at the same time, and the good psychometric properties cannot automatically be supposed to also be valid in this sample, where each item was answered on a separate day. We did omit 1 item: ‘felt giddy.’ This was due to low reply frequencies, which means that the scale presented was incomplete.

Another limitation is that approximately 350,000 people logged in on a given day, raising the possibility of selection bias. Due to the fact that the subjects were anonymous, we could not investigate selection effects. However, a recent study using the Internet for health-related topics was independent of gender, age, and diagnosis in a group of patients with psychosomatic disorders [60]. Additionally, the response rate obtained at LunarStorm was very high for such a generalized Internet-based survey. Data were sampled in 2005, and it is possible that response patterns could have been different, if the survey would have been launched in 2016, considering, for example, the global economic crisis that has emerged during this time span. Societal and environmental changes as well as changing attitudes toward psychosomatic symptoms, increasing Internet access, and the readiness to report complaints on a Web community could be some of the factors, which might have had an impact on response patterns.

Because studies on Internet-based assessment are a relatively recent phenomenon, the validity of the data gathered in this manner is uncertain and worth further exploration. In our study, the spatial resolution is low and areas with some of the highest variability in socioeconomic factors were merged together by the LunarStorm site administrator and classified as “major city area.” Because perceived stress and ill health have a strong association with socioeconomic status, the merging of areas might have attenuated our findings. A higher spatial resolution might have shown more pronounced differences between areas, also because within city areas there are large socioeconomic and neighborhood differences [61].

### Strengths

One of the strengths of the present study is that the subject could log into her/his own LunarStorm corner privately and at a suitable time, and voluntarily chose to participate. While representing a convenience sample, this raises the probability of sincere replies.

Another advantage is the 100,000 to 130,000 of subjects of various ages who responded. The received responses from 100,000 to 130,000 individuals per day, represents on average 36% of the entire population of members logging in daily (n=360,000). This volume of responders would be difficult to reach in such a short time space by other ways of communication. Furthermore, such administrative factors as data transcription, the risk of excluded values and ‘odd’ answers, and the concern that other people might read the answers can be overcome by computer- and Internet-based surveys.

### Conclusion

Young people in Sweden have a generally high prevalence of self-reported psychosomatic complaints, and these seem to be more common in major city areas as compared with minor city/rural areas. As urbanization progresses globally this might be of importance as a risk factor hampering the wellbeing of children and adolescents. The current study provides valuable information on the importance of regional differences and the potential benefit of living closer to nature, which should be taken into account when planning for healthier living environments. The study inspires to identifying urban environmental features that promote health as well as finding interventions to raise subjective and collective psychological resilience of, especially young, city dwellers. In addition, it motivates further studies exploring the causality and mechanistic explanations for environmentally as well as socioeconomically related links to psychosomatic health.

## Abbreviations

PBS: psychosomatic problems scale
